# Consolidating and Upscaling Molecular Research Capacity in Nigeria: On Who's Account?

**DOI:** 10.3389/frma.2021.788673

**Published:** 2022-01-05

**Authors:** Chinwe Uzoma Chukwudi

**Affiliations:** Department of Veterinary Pathology and Microbiology, University of Nigeria, Nsukka, Nigeria

**Keywords:** molecular research, funding, research capacity, research output and impact, Nigerian economy

## Abstract

Molecular research and researchers engage in studies that seek to understand the structures, functions, and interactions of biomolecules as the basis for cellular and systemic effects in living organisms. This research approach was made possible by considerable technological advancements that equip researchers with tools to view biomolecules. Although molecular research holds great promises for improving lives and living, the technological requirements and equipment to undertake molecular research are quite expensive, often requiring a heavy start-up capital or investment. In developing countries such as Nigeria, where the majority of the population lives below the poverty line and research funding is abysmally low, such heavy investments into research that do not provide immediate solutions to societal problems are difficult. This is mostly due to limited resources available to tackle many urgent and pressing needs, and limited perspective and understanding of policymakers, leading to infrastructural and skilled personnel deficit to support molecular research. Despite all these, the field of molecular research continues to grow exponentially globally, hence, funding and investments into this critical life science research area have become imperative. With the rich biodiversity of humans, animals, and plants in Nigeria, and the huge burden of infectious diseases in the country or region, global advances in genomics and proteomics studies will be incomplete without adequate contribution from Nigeria and sub-Saharan Africa region. This paper examines the progression and challenges of undertaking molecular research in Nigeria, and how Nigerian molecular research scientists are tackling these issues, with recommendations for improved molecular research capacity and output in the country or region.

## Introduction

Molecular biology is a branch of life science that studies the structural and functional aspects of the characteristics and interactions of the molecules that form the basis of life (Morange, 1999). These molecules include DNA, RNA, and proteins, which have similar characteristics in all forms of life, whether microbes, plants, animals, or humans. Hence, molecular research is the area of research that focuses on these biomolecules, in contrast to the previously practiced study of life at the level of cells, tissues, organs, and systems (Astbury, 1961; Chukwudi, 2016). The fields of molecular research studies include genomics—the study of genes (mostly DNA, and sometimes RNA in viruses), transcriptomics—the study of messenger RNAs, proteomics—the study of proteins, and metabolomics—the study of small molecule biochemical metabolites (Vailati-Riboni et al., 2017). Advances in molecular research have led to improved plant and livestock breeds for improved food production (Loor, 2010; Bai et al., 2012), improved understanding of the genetic and epigenetic basis of human and animal phenotypes and diseases (Fong et al., 1998; Van Lier et al., 2010; Marchant et al., 2014), and improved understanding of microbial agents in the fight against infectious diseases (Tang and Procop, 1997; Emmadi et al., 2011). For instance, it is because of advances in molecular research that COVID-19 diagnosis and treatments or vaccines could be developed in such record time (Habibzadeh et al., 2021). Most of the times (as in the case of COVID-19), the benefits of molecular research become evident following long-term investments and diligent studies. The Human Genome Project has been going on for several years, but its contributions to improved health and disease management are only beginning to be felt (Khan et al., 1998; Hood, 2002). In medicine, molecular research holds future prospects of personalized medicine and gene therapy (Hamburg and Collins, 2010).

These contributions of molecular research have been made possible by technological advances that enable scientists to view and study these subcellular components and molecules in living organisms (Hood, 2002; Chandramouli et al., 2009). Genes could be studied because researchers could extract DNA from organisms and explore their characteristics, relationships, and interactions (Lee et al., 2012), same with RNA and proteins (Walter et al., 2002; Josefsen and Nielsen, 2011; Mahmood and Yang, 2012). To undertake molecular research, scientists require cutting-edge equipment and specialized reagents for the study of biological molecules (Voelkerding et al., 2009; May et al., 2011). However, these facilities (equipment and reagents) do not come cheap, hence are not readily available to scientists or researchers without adequate research funding.

Nigeria is a country with a rich biodiversity—humans, animals, and plants (Osawaru et al., 2013; Federal Republic of Nigeria, 2015), and also microbes (Izah et al., 2018; Anwadike, 2020). With an estimated population of over 200 million people (projected to double by 2050), and a population density of 167.5 people per square kilometer, Nigeria is considered the most populous country in Africa (accounting for about 19% of the continent's total population), the second fastest growing population and the seventh most populous country in the world (Akinyemi and Isiugo-Abanihe, 2013; Reed and Mberu, 2013; United Nations, 2019a,b). With the interconnectedness of human health, animal health and environmental health (Staudinger et al., 2013; WHO, 2015; Mills et al., 2019), and the complex evolutionary trends of microbial genetic materials across host organisms (Lohbeck et al., 2016; Chiu and Miller, 2019), global efforts at genetics and molecular health research will be incomplete without contributions from the rich biodiversity of Nigeria. However, funding for genetic and molecular research in Nigeria is abysmally low to meet the needs of such a country of rich diversity. This is largely due to the dwindling economy of the country, with ever-increasing needs and limited resources, and a generally poor prioritization of research by the government. This paper assesses the challenges of upscaling molecular research in Nigeria and their implications, with a view to identify strategies for improved productivity in this regard.

## The Current Outlook and Challenges

With the rich natural and human resources available in Nigeria (often referred to as “the Giant of Africa”), one would expect the country to take the lead in molecular life science research. At the beginning of the COVID-19 pandemic, Nigeria demonstrated such leadership in Africa as a testament to her enormous molecular research capability by sequencing the SARS-CoV-2 genome from the index case within 48 h of sample submission (Makoni, 2020; Paul, 2020). Subsequently, more work have emerged from Nigeria contributing to the tracking of SARS-CoV-2 variants and mutations (Awoyelu et al., 2020; Happi et al., 2020; Shaibu et al., 2021). However, the volume of molecular research output emanating from the country is far below the country's potential capacity and expectation, given her enormous human resources and biodiversity. Several economic factors contribute to this low productivity, as outlined below.

### Equipment and Reagents

It is quite apparent that molecular research is driven by technological innovations and inventions that are constantly evolving, hence requiring state-of-the-art facilities. Molecular research equipment is often sophisticated and requires specialized reagents with strict cold storage conditions. Not many small research laboratories in Nigeria can afford these cutting-edge facilities. However, a few specialized laboratories that are quite well-equipped exist and often serve as national reference laboratories. These include the laboratories at Nigerian Institute of Medical Research (NIMR), Nigerian Veterinary Research Institute (NVRI), Nigeria Centre for Disease Control (NCDC), African Centre of Excellence for Genomics of Infectious Diseases at Redeemer's University, Ede, 54 gene laboratories, etc. Most of these laboratories with functional molecular research capacity were either established, and/or sustained by foreign or international fundings and partnerships, or private sector intervention (Folarin et al., 2014; Maxmen, 2021). Nevertheless, these are not only too few to meet the molecular research needs of such populous country as Nigeria, but are also far in-between. Hence, samples for molecular research and diagnostics have to be transported over long distances and time interval from the sites of collection to these few laboratories with functional molecular research capacity, often leading to sample degradation and loss of integrity (Luka et al., 2011; Nwagbo et al., 2016; Ugochukwu et al., 2021). The integrity of both samples and reagents is not guaranteed with the epileptic power supply obtainable across the country. At best, some of these so-called reference laboratories are only able to perform basic molecular techniques; hence, more serious techniques are often sent to foreign collaborators who have the capacity to apply more sophisticated molecular techniques (Omar et al., 2017; Pitt et al., 2018; Onyiche et al., 2020). This is usually done at huge shipping or travel costs to the researchers.

### Institutional and Policy Support

In recent times, a lot of small research laboratories are beginning to acquire bits and pieces of basic molecular research equipment, seeing that they really cannot do much without them. But where and when they do, the challenge of expensive and inaccessible reagents has continued to mitigate such efforts. These efforts are greatly hampered by a general lack of support from both the institutions and government policymakers. Many research institutions or universities do not have a functional central research administration office that could help researchers and research laboratories procure the requisite equipment and reagents. Hence, individual researchers and laboratories are left to source these facilities on their own, often making their efforts unnecessarily cumbersome as many of the molecular research companies (which are mostly foreign) are not willing to deal with individuals. With great determination, a few researchers are able to break through the companies' bureaucratic protocols, only to be stalled by unfavorable government policies that further exacerbate their difficulties. Because there is no clear working policy on the importation of research equipment and reagent (in addition to the apparent lack of institutional support), these researchers end up importing equipment and reagents that are eventually left to damage or deteriorate at the ports due to overbloated import duties (billed to these researchers) and exploitation by corrupt and/or ignorant port officials. In view of all these, many researchers and small laboratories rely on business men who know their way around with importation for their equipment and reagent needs. Some of these businessmen (often with no form of scientific or research training) have few or no knowledge of the conditions of storage for these equipment and reagents. Sometimes, they can compromise on quality to maximize their profits. Hence, some researchers end up buying substandard equipment and degraded reagents from them, which would in turn adversely affect their research productivity. Consequently, there are many small laboratories with some basic molecular research equipment, but which are still largely not functional (or functioning below capacity) due to inaccessibility of (or inadequate access to) reagents required to run the equipment.

### Skilled Personnel

In addition to specialized equipment, molecular research also require specialized skills and adequately trained personnel. In the earlier days of molecular research in Nigeria, one of the major challenges was that of having adequately trained manpower. However, as molecular research capacity building efforts continue to improve, many Nigerian research personnel have received specialized molecular research training (mostly at foreign institutions), and many have returned home after their training to help to build molecular research capacity in their home institutions. Unfortunately, the challenges of non-functional laboratories and poor or unconducive working environment are now driving the emigration of such skilled personnel to the technologically advanced countries where they would be more productive (The Royal Society, 2011). Besides being largely unproductive or underperforming due to the generally perceived unavailability of molecular research facilities in Nigeria, these highly skilled personnel are often poorly remunerated. The salaries of Nigerian academics and researchers could be as low as a 10th of that of their counterparts in more advanced countries (Tade, 2020), albeit with a huge disparity between those in government-owned institutions and those in private institutions. With very few opportunities for research funding, conference attendance, and publication fees for the huge number of researchers, many of these researchers resort to funding their research, conferences, and publications from their meager remunerations for their career progression and promotions. Under these frustrating and limiting conditions, and with the incessant trade disputes between the Nigerian government and academic researchers, many of these highly skilled molecular researchers are forced to seek employment in other countries in search of greener pastures (Adeloye et al., 2017; Isbell and Ojewale, 2018). Hence, a critical mass of the molecular research potential emigrates (Adebayo and Akinyemi, 2021; Maxmen, 2021) leaving behind mostly those who have the capacity to engage in other ventures to augment their earnings. The distraction that comes with trying to augment the poor research remunerations ultimately hinders these remaining researchers from giving the requisite or full attention to their research, leading to a further decline in molecular research capacity and output.

### Funding

Generally speaking, research funding in Nigeria is very poor. In addition to the issues of equipment or reagents and personnel remunerations (which are also indirectly related to funding), direct research funding is very limited (Osagie, 2012). Apart from the sparingly available foreign research funding, local research funding is abysmally low or few in Nigeria (Bello, 2012; Baro et al., 2017). Hence, most research works emanating from researchers in Nigeria are personally funded by the researchers themselves from their meager salaries (Baro et al., 2017). How much of and the quality of molecular research (with its very expensive reagents and equipment) that could be personally funded from an individual's pocket could then be imagined. Nevertheless, the federal government of Nigeria attempts to provide some “incentives” to stimulate research *via* the Tertiary Education Trust Fund, TETFund (Olayiwola, 2010; Baro et al., 2017). The entire federal government funding for education is bundled into TETFund, which was set up as an intervention agency with the main objective to manage, disburse, and monitor the education tax funds to public tertiary institutions in Nigeria for the provision and maintenance of essential physical infrastructure and materials or equipment to promote teaching and learning, research and publications, and human capacity development (academic staff training and development). Additionally, TETFund is to achieve all of this with 2% assessable limited liability company profits collected as education tax funds in the country. By the time the funds are spread over all the mandates of TETFund (which is the “largest holder of research grants in Nigeria”), Nigeria celebrates a total government research funding of 7.5 billion in 2020 (about $13.4 million at the current exchange rate of 560/$1) for all the public universities serving a population of over 200 million people (Adedigba, 2020). Comparatively, in 2017, the US government invested $39.5 billion in medical and health research and development alone, which is just about 5% of total health spending (Schwartz and Woloshin, 2018). This was still considered inadequate, despite additional industry investment of $121.8 billion in medical and health research. At the end of the day, Nigeria's total research budget comes to about 0.2% of her gross domestic product (Maxmen, 2021), and the TETFund allocations for molecular research are too meager to make much meaningful impact. Not only is the public research funding stretched thin in Nigeria, accessibility of the funds by the researchers is a herculean task (even when a researcher has been awarded the research grant), and this places a great constraint on research productivity (Olayiwola, 2010; Fosci et al., 2019). A government that does not fund research cannot set research agenda, and most incredibly or unfortunately, the Nigerian government argues that “government funding will flow more freely once science starts to deliver tangible benefits” (Maxmen, 2021). Who then will give the researchers what they need to deliver tangible results? Conceivably, they have to and are already doing it from their own pockets (with their meager salaries) from the actual research costs to conference attendance and publication fees.

## Implications and Impact on Research Output

The circumstances described above have various implications, and impact on molecular research activities and output in Nigeria. Some of these include:

### Aversion

With all these impediments bedeviling the research and development (R&D) sector in Nigeria, and the added peculiar challenges of conducting or engaging in molecular research in such difficult terrain, many Nigerian researchers try to avoid or circumvent molecular research by all means possible. Since the laboratory facilities for conducting molecular research are not readily available, even those trained or highly skilled in molecular research will rather do any other kind of research to be able to meet up with the requirements to survive in their careers. Hence, most of these molecular researchers get engaged in other areas of research away from their core competencies and interests in a bid to keep their careers afloat. Even when they conduct seemingly molecular research, they carefully design it in such a way as to evade the core molecular investigations, or merely scratch the surface of what should have been an in-depth molecular investigation (Ugochukwu et al., 2021). At best, many resort to computational genomics, data mining, bioinformatics, and other *in silico* research (which they could do with only their laptops), instead of generating primary genomics data from the abundant microbes or applying their skills in investigational laboratory and/or diagnostics settings (Awoyelu et al., 2020). Some others completely move away from the area of molecular research and find other research interests for themselves as a survival mechanism. Downstream, this discourages upcoming researchers from pursuing training and career in molecular research, thereby worsening the inadequacy of skilled personnel for molecular research. As a junior colleague or mentee who was being encouraged to undertake training in molecular research at a foreign institution succinctly put it, “I want to train in an area that will be useful to me when I return.” This is because those who had previously received such training are generally perceived as “useless” to their home institutions and incapable of generating reasonable research output once back home (The Royal Society, 2011). Hence, they become the subject of ridicule among peers, and examples of “what not to be” for aspiring researchers, instead of being mentors of molecular research skills to the upcoming generation of researchers and molecular research capacity builders for their institutions and country.

### Few and/or Low-Quality Molecular Research Output

When researchers manage to conduct molecular research in Nigeria, the output is generally low (compared with the potentials), and often deficient in quality. Without adequate equipment and reagents, generating high-quality data and verification of results is quite difficult. Because molecular research reagents are expensive and inaccessible to these researchers, conserving what they have to maximize productivity becomes paramount, often overruling the need to optimize procedures to generate high-quality or reproducible data. Such scarcely available reagents must be preserved for when they are absolutely indispensable, and not for “playing around” in the laboratory (which is actually what “experimenting” is about). In a bid to conserve resources, some of these reagents are kept and used even beyond their shelf lives, since a replacement may be far-fetched. Similarly, some molecular laboratory equipment available in some institutions are never fully put to use before they become obsolete. Hence, these molecular researchers in Nigeria are stuck with obsolete equipment with difficult or laborious protocols even when more sophisticated equipment and easier methodologies are available (but unfortunately inaccessible or unaffordable). For the greater majority of the researchers who do not have immediate access to molecular research facilities, the challenges of sample preservation with epileptic power supply and transportation to the few equipped (specialized or reference) laboratories often predispose their samples to degeneration, thus further complicating their studies (Ugochukwu et al., 2021). Consequently, these low-quality data are rarely publishable in high-quality journals, thereby worsening the dearth of molecular research data from Nigeria as many of these research works end up on the shelves of the researchers (despite all the effort they put into it). Those who manage to get such studies published often do so in low-impact journals with low visibility, as they would not be able to meet up with the review requirements of the high-impact and high visibility journals (which may ask for a revalidation of some of the results, something the unfunded researcher cannot afford). Even when the research output is considerably high quality and acceptable in high-impact journals, these self-funded researchers often cannot afford the high publication fees (which may be as high as their annual salaries). Therefore, these research studies still end up in low-impact and poorly visible journals, thereby further exacerbating the dearth of molecular research data. This in turn increases the aversion of researchers to molecular research in Nigeria.

### Attribution of Indigenous Intellectual Property to Developed Countries

It is important to note that the lack or scarcity of molecular research data and output from Nigeria does not in any way represent a lack of ideas from the researchers. As a matter of fact, many brilliant and innovative research ideas abound in the conceptual existence of the molecular researchers in Nigeria. However, for many of these research ideas to see the light of day, much of the actual laboratory work and data generation have to be done in foreign institutions with requisite equipment. Sometimes, to have access to these foreign institutions and their equipment, these Nigerian researchers use the little amount of funds they are able to attract or generate to travel to these foreign institutions and carry out their work. Most annoyingly, the only internal funding agency of the government (TETFund) allocates a greater proportion of the meager resources available for academic staff training and development to foreign training and bench work (60%), and the beneficiaries take the funds to travel to a foreign institution and work on their ideas. At the end of the day, the research output and intellectual properties accruing to such studies are attributed to the foreign institutions, whereas the researchers return to their home institutions with their “paper” qualifications (certificates), and no laboratory equipment to continue the work for which they had been trained. Some other researchers have to find collaborators to give their work the “touch of class” it needs to become internationally acceptable and/or publishable. With a general dearth of intellectual property and technology transfer skills or support in many Nigerian institutions, some of these collaborators end up exploiting the “helpless” researchers, and taking credit for the research output generated. Consequently, several research publications, innovations, inventions, and patents that could have accrued to Nigeria and Nigerian institutions have in this way been attributed to other countries with state-of-the-art research facilities where these studies and inventions were further developed, as the Nigerian inventors or researchers could not develop them in the country. This forms a vicious cycle that keeps impoverishing the country intellectually, infrastructurally, technologically, and economically.

### Loss of Funds and Productive Time

Apart from spending a huge proportion of their budget and time on foreign travels to get most molecular research work done, Nigerian researchers pay a lot to ship samples, and reagents from other countries. Many times, these items get held up at the ports, become degraded, damaged, or completely lost. In addition to the loss of the funds used to ship and reship these samples and reagents, a lot of time that would have been used for the actual study is lost in the sample preparation and shipping duration. This process may often have to be repeated over and over until the samples or reagents are received in good condition for the study to make progress. Hence, the very limited funds are further wasted, and work that would have taken days or weeks (had the necessary equipment and reagents been available) end up taking months and years to complete. In the case of sample loss during shipment or storage, the sampling procedure will have to be started afresh, leading to more loss of productive time and resources (which is why many will rather spend the money to travel with their samples if they can).

### Knowledge Gap or Disconnection of Research From Reality

With the rich biodiversity in Nigeria, global research efforts in the area of human, animal, plant, and microbial genomics will be incomplete without adequate contributions from Nigeria. In particular, global efforts to combat infectious diseases would be grossly inadequate or misdirected without appropriate contributions from researchers who have first-hand experience of the realities of these disease conditions. Researchers from developing countries such as Nigeria live and work in the midst of the numerous infectious disease burdens, but are limited by unavailability of facilities and resources from instituting appropriate scientific research investigations and interventions. Conversely, many of the researchers in the developed countries who have the facilities to undertake molecular research to combat these diseases have no practical experience of the diseases, and therefore, have no understanding of the practical realities and challenges of the conditions they are researching into. Hence, much of their research are more theoretically inclined, instead of having practical implications. Some are so disconnected from the realities of the disease conditions that they are still using samples and data from several years ago to make research decisions for conditions and pathogens that have evolved over time in the endemic areas. It takes an understanding of the real-life situations or conditions to connect research to reality by making informed research decisions, such that the research strategy would be more targeted at offering practical and appropriate interventions. For instance, some vaccines have been developed that could not offer protection against local strains of the pathologic microbes (Saravanan et al., 2010; Ukwueze et al., 2020), often because these local strains are unknown to the vaccine developers. The efforts put into such vaccine development process could have been more efficacious if the vaccine developers received the contributions of local researchers to take into consideration the genomic compositions of the local strains (Luka et al., 2011). These local researchers may also be able to proffer a more effective intervention if adequately empowered to undertake molecular research.

### Lack of Research Agenda or Focus and Specialization

The tenacious molecular researchers who continue to work under these limiting conditions can hardly focus on an area of specialization, if they must progress in their career. Many times, instead of concentrating on their core areas of interest and specialization to improve on them, they go on to engage in any kind of research that will enable them to acquire the basic requirements needed for their promotions and career progression. This robs them of the joy of doing what they love or want to do, thus depriving them of job satisfaction and the internal motivation that comes with it. In addition, it also robs them of the opportunity to become better in their chosen fields of molecular research through constant practice and engagement. This further aggravates the aversion to molecular research and poor molecular research output, thereby undermining efforts at building or increasing the molecular research capacity of Nigerian institutions.

## Strategies for Upscaling Molecular Research Capacity and Output

In view of the enormous challenges outlined above, and the negative impact they have on the quantity and quality of molecular research output in Nigeria, there is an urgent need to improve the molecular research capacity of Nigerian institutions and researchers. This is essential not only for the purpose of improving molecular research output from Nigeria, but also will accord the global research community the benefits of the invaluable contributions from the rich biodiversity and innovative intellectual potentials of the country in tackling global health challenges. All hands must be on deck for this vicious cycle of dwindling molecular research capacity and output to be terminated. Let us consider some of the strategies that could be employed by the various stakeholders to achieve the much-desired improvement.

### Researcher Led

Although the working conditions are far from being ideal, molecular research scientists in Nigeria must find an inner motivation to remain committed and resolute, knowing that their work will greatly impact their local communities. They need to stay focused on the goal of solving problems, even though that comes with a lot of personal discomfort. If they all should emigrate to look for “greener pastures,” who would fill the knowledge gap and supply the local content that is necessary to bring appropriate research-driven solutions to the local challenges? As they are already doing, they should continue to collaborate with both local and foreign colleagues to achieve their research aims. However, it is important for them to receive training in technology transfer to avoid being exploited and their intellectual property deprived in such collaborations. Equipment sharing with colleagues within and among institutions would be beneficial to all, and efforts should be made to create a national database of functional cutting-edge equipment and grant easy access to those who need them for their research. Also, instead of using their limited funds to travel to foreign collaborating laboratories to do work, they could encourage these foreign collaborators to “adopt” their local laboratories and help them to acquire some requisite equipment and reagents. They should also do their best to maximize the sparsely available local and foreign research fundings to showcase their potentials, thereby setting themselves up to be able to attract more funding. Although they may not be able to afford the open access publication fees of most high-ranking journals, they could always publish their work in the few good journals that require no or low publication fees.

### Institution Led

The research institutions need to step up efforts to provide the necessary support to the researchers by providing a conducive working environment for them. Such basic amenities and services as office and laboratory space, reliable power and water supply, waste disposal, and generally improved infrastructure are critically lacking and should be tackled more seriously by the institutions, so that the researchers could concentrate on doing research. Where necessary, the institutions should explore public–private partnerships to develop and manage the requisite infrastructure. Institutional research management and grant management offices are desperately needed to help the researchers with procurement of equipment and reagents for their work, and also manage their grants effectively without additional administrative burden to the researchers. This will in turn improve the institutions' standing with funding agencies and attract more grants. The concept of central and regional laboratories should be pursued within and between institutions to ease researchers' access to specialized equipment. Institutional technology transfer offices should be established and/or strengthened to assist researchers negotiating a fair deal during technology transfer agreements with collaborators and protect their intellectual property.

### Government Led

Although Nigerian institutions are making efforts to provide basic infrastructure such as power supply, the problem of unreliable electricity supply and dilapidated physical infrastructure affects the whole country and can only be resolved by the government. It is high time the government steps up with a definite research policy and agenda setting to give research direction to researchers. This cannot be achieved without the government prioritizing research and improving research funding. There is an urgent need to reassess TETFund allocations to research funding and restructure appropriately to reflect the research needs of the country. Also, the government needs to enhance the ease of doing research business in Nigeria with policies that will ease the financial and administrative burden of importing research equipment and reagents.

### Funding Bodies and Agencies

Given the critical need to harness Nigeria's (and Africa's) rich biodiversity for global good, and in the light of the peculiarities of conducting molecular research in developing countries such as Nigeria, funding bodies need to increase specifically targeted molecular research funding to researchers living and working in these countries. These researchers need to be motivated and adequately empowered to fill the molecular research gaps in their localities. More emphasis should be placed on equipping the local laboratories with modern facilities to conduct meaningful and in-depth molecular research locally. To this end, the National Institutes of Health (NIH), in partnership with Wellcome Trust, initiated a 10-year research funding (2011–2021) for the Human Heredity and Health in Africa (H3Africa) project to support African institutions for biomedical research projects and empower African researchers to be competitive in genomic sciences. This project has recorded a massive success, supporting about 51 projects in 30 African countries, and training about 405 PhDs, 407 MScs, and 222 BScs in genomic sciences on the African continent (https://h3africa.org). Once again, the NIH, in partnership with Bill and Mellinda Gates Foundation and the African Academy of Science, is taking the lead with the African Postdoctoral Training Initiative (APTI), which provides training and research funds at home institutions for African postdoctoral researchers. More of such funding is crucial to fill the desperately needed molecular knowledge gap and build the requisite molecular research capacity in Nigeria and Africa in general. Also, international conference organizers and journal publishers would be doing a great service to the scientific community by granting fee waivers to these unfunded or underfunded molecular researchers who, against all odds, have something to show for their research efforts. This would give them the platform to communicate their findings to the rest of the world and lend their voices to pertinent molecular research issues with no extra burden on their already lean pockets.

## Conclusion

This study examined the current outlook of molecular research in Nigeria, while outlining the numerous challenges faced by the indigenous scientists undertaking molecular research in Nigeria. The implications and impacts of these challenges on the molecular research output from Nigeria were discussed, and also some strategies for upscaling molecular research capacity and output in the country. The implementation of these strategies by research policymakers and funding bodies would empower indigenous researchers to employ molecular technologies to proffer solutions to indigenous problems, thereby reforming molecular research in Nigeria. This could serve as a springboard for the evolution of molecular research in Nigeria in particular, and Africa in general.

## Author Contributions

The author confirms being the sole contributor of this work and has approved it for publication.

## Conflict of Interest

The author declares that the research was conducted in the absence of any commercial or financial relationships that could be construed as a potential conflict of interest.

## Publisher's Note

All claims expressed in this article are solely those of the authors and do not necessarily represent those of their affiliated organizations, or those of the publisher, the editors and the reviewers. Any product that may be evaluated in this article, or claim that may be made by its manufacturer, is not guaranteed or endorsed by the publisher.
